# EXPLORING OLDER ADULTS’ PERCEPTIONS OF PRESENCE AND IMMERSION IN DIVERSE VIRTUAL ENVIRONMENTS

**DOI:** 10.1093/geroni/igz038.895

**Published:** 2019-11-08

**Authors:** Walter R Boot, Andrew Dilanchian, Ronald Andringa

**Affiliations:** Florida State University, Tallahassee, Florida, United States

## Abstract

Virtual Reality (VR) holds great promise for enhancing the health, well-being, and skills of older adults. However, VR solutions must consider the age-related “digital divide;”many older adults have less experience and proficiency with a number of newer technologies, which may serve as a barrier. Older adults especially have less experience with virtual environments, an experience many younger adults have acquired through video gaming. This study compared younger and older adults’ perceptions of immersion and presence in a series of diverse virtual environments using the HTC Vive. Participants experienced a VR meditation task, “indoor" and “outdoor” navigation tasks, and a fast-paced action game. Importantly, younger and older adults reported similarly high experiences of immersion and presence within virtual environments, and contrary to expectations, older adults reported fewer symptoms of cybersickness. Results suggest VR as a promising tool to promote the health and well-being of older adults.

